# Commercial scale production of Yamabushitake mushroom (*Hericium erinaceus* (Bull.) Pers. 1797) using rubber and bamboo sawdust substrates in tropical regions

**DOI:** 10.1038/s41598-023-40601-y

**Published:** 2023-08-16

**Authors:** Preuk Chutimanukul, Wongsakorn Phatthanamas, Ornprapa Thepsilvisut, Thanin Chantarachot, Akira Thongtip, Panita Chutimanukul

**Affiliations:** 1 Department of Agricultural Technology, Faculty of Science and Technology, Thammasat University, Rangsit Centre, Khlong Nueng, Khlong Luang, Pathum Thani, 12120 Thailand; 2 Department of Botany, Faculty of Science, Center of Excellence in Environment and Plant Physiology, Chulalongkorn University, Pathum Wan, Bangkok, 10330 Thailand; 3 National Center for Genetic Engineering and Biotechnology (BIOTEC), National Science and Technology Development Agency, Khlong Luang, Pathum Thani, 12120 Thailand

**Keywords:** Fungi, Biotechnology

## Abstract

Yamabushitake (*Hericium erinaceus*) is one of the most sought out mushrooms that is widely used for both direct consumption and medicinal purposes. While its demand increases worldwide, cultivation of the mushroom is limited to temperate areas and its production in tropical regions has never been explored. The aim of this study was to test the utilization of rubber and bamboo sawdust, alone or as a substrate mixture, for industrial scale Yamabushitake mushroom production. Five substrate treatments with various ratios of the two sawdust were compared for their physicochemical properties in relation to mushroom productivity. The highest mushroom fresh and dry (113.22 and 23.25 g, respectively), biological efficiency (42.61%), and cap size (9.53 cm) were obtained from the substrates containing 100% rubber sawdust, with the mushroom yield decreasing proportional to the ratio of bamboo sawdust. The 100% rubber sawdust substrate provided a higher initial organic matter and carbon content together with C:N ratio at 63.2%, 36.7% and 65.48, respectively, whereas the 100% bamboo sawdust provided higher nitrogen content (1.03%), which was associated with lower mushroom yield but higher number of fruiting bodies. As in the 100% rubber sawdust substrate, a comparable mushroom yield and growth attributes were also obtained in the 3:1 rubber-bamboo sawdust mixture substrate. Principle component analysis of the measured variables indicated a strong influence of substrate C:N ratio before spawning and the change in substrate electrical conductivity and N content after cultivation to the variation in mushroom productivity among the treatments. The results demonstrate the applicability of rubber sawdust and its combination with up to 25% of bamboo sawdust for Yamabushitake mushroom cultivation and provide the basis for substrate optimization in the tropical Yamabushitake mushroom industry through a circular economy framework.

## Introduction

*Hericium erinaceus*, also known as Yamabushitake or Lion’s Mane, is an edible mushroom that has been recognized in traditional Asian medicine for its nutritional and medicinal qualities. It is also considered functional food due to the presence of high fiber content and essential amino acids^1^. On a dried weight basis, Yamabushitake fruiting bodies contain approximately 6.3–77.5 g of total sugar^2–4^ and 4.3% reducing sugar^5^, including such beneficial polysaccharides as β-glucans, α-glucans and glucan-protein complexes^3,6^. Pharmacological studies have indicated the benefits of Yamabushitake polysaccharides in preventing, treating, and alleviating some diseases, including cancer, gastric ulcers, diabetes, hyperlipidemia, hepatic damage, and neurodegenerative diseases^7–12^. The bioactive ingredients conferring these effects could be further isolated and developed into a pharmaceutical formulation. Due to such nutritional and medicinal properties, there is an increasing demand for the mushroom from both direct consumers and the healthcare industry, providing an economic opportunity for farmers worldwide.

Yamabushitake is one of the five most popular cultivated mushrooms in the world^13^, with Japan as the world leader in its production, followed by China, South Korea, Taiwan, USA, and Canada^14–16^. As it originates from a temperate region, the mushroom requires low temperature of 18–24 °C to produce fruit bodies^17^. While the consumption of Yamabushitake mushroom has expanded globally, its main production areas remain in the temperate regions. In fact, industrial Yamabushitake mushroom production has received relatively little attention in tropical regions, despite the fact that millions of tons of lignocellulosic materials from agricultural and agro-industrial sectors are readily available and wasted annually^18^. These materials can potentially be used as low- or zero-cost substrates for Yamabushitake mushroom cultivation. To fill this gap, there is a need to develop a system for Yamabushitake mushroom production in the areas, particularly in the light of promoting the circular economy where some lignocellulosic agricultural byproducts and agro-industrial wastes can be turned into high-value products^19,20^.

In Thailand, the production of edible mushrooms mainly relies on agricultural wastes, such as sawdust and rice straw, to make them more commercially viable^21,22^. Generally, commercial-scale mushroom production in the country utilizes rubber sawdust as the base substrate, but whether this is applicable for Yamabushitake mushroom has never been explored. Nevertheless, there can be a shortage of rubber sawdust due to limited seasonal availability and competition from other industries, which could pose a production problem to the mushroom farmers. It is thus also necessary to find an alternative substrate to replace rubber sawdust to overcome this challenge. In Thailand, bamboo can grow anywhere, and at least three tons of bamboo sawdust are generated each day by the local industries, most of which is disposed of by incineration, causing environmental pollution^23^. As bamboo sawdust contains the cellulose, hemicellulose and lignin profile similar to conifer sawdust^24^, it has a potential to be used as a growing substrate for edible mushrooms. It can also be an alternative substrate for Yamabushitake mushroom cultivation and may serve as a substitution for rubber sawdust when needed.

This study aimed to test at a commercial scale the feasibility of using rubber sawdust for Yamabushitake mushroom cultivation, and to determine whether bamboo sawdust can be used as an alternative substrate for Yamabushitake mushroom production. To this end, five substrate treatments containing rubber and bamboo sawdust, individually alone or as a mixture at different ratios, were compared for their physicochemical properties and applicability to promote the mushroom growth and yield. Our data provide a framework for the utilization of rubber and bamboo sawdust for commercial Yamabushitake mushroom cultivation in tropical countries.

## Materials and methods

### Study area and mother culture

The study was carried out between October of 2018 and March of 2019 at Fresh & Friendly Farm Co., Ltd (13° 59 30.2 @N, 100° 38 18.2 @E) in Pathum Thani province, Thailand. Yamabushitake mushroom spawn (*Hericium erinaceus* (Bull.) Pers. 1797) was supplied by Fresh & Friendly Farm. The laboratory analyses were performed at Major of Agricultural Technology, Faculty of Science and Technology, Thammasat University, Pathum Thani, Thailand.

### Substrate preparation and mushroom cultivation

Rubber sawdust was purchased from Fresh & Friendly Farm in Pathum Thani, Thailand. Sangmon bamboo stems (*Dendrocalamus sericeus* Munro) were obtained from a bamboo garden (14° 30 37.2 @N, 100° 01 17.2 @E) in Suphan Buri province, Thailand. The bamboo was chipped into sawdust with a wood chipping machine (Model 2233 HS-18, Patipong Industry, Thailand) at Major of Agricultural Technology, Faculty of Science and Technology, Thammasat University. Other raw materials were purchased from a local market in Pathum Thani, Thailand. The cultivation substrate was formulated based on the existing commercial mushroom substrate, containing rubber sawdust: corn cob: rice bran at the ratio 3:1:1 on a dry weight basis (150 g rubber sawdust, 50 g corn cob, and 50 g rice bran). To determine whether bamboo can be used as a substitute for rubber sawdust in the substrate for Yamabushitake mushroom production, five sawdust ratio treatments were prepared: T1 was sole rubber sawdust (100%RT); T2 was rubber sawdust mixed with bamboo sawdust at the ratio 3:1 (75%RT:25%BT), T3 was rubber sawdust mixed with bamboo sawdust at the ratio 1:1 (50%RT:50%BT), T4 was rubber sawdust mixed with bamboo sawdust at the ratio 1:3 (25%RT:75%BT), and T5 was sole bamboo sawdust (100%BT). Each of these sawdust substrates was then mixed with corn cob and rice bran at the ratio 3:1:1 on a dry weight basis. The pH of mushroom substrates was adjusted with lime (Ca(OH)_2_) to 6 ± 1 with 65% relative humidity (RH). For mushroom cultivation, a bottle was filled with 750 g of the substrate on wet weight basis.

After sterilization, the growing media was inoculated with the spawn of 30 sorghum grains and incubated at 20 °C in the presence of light for mycelial colonization. After seven days of incubation, the bottles were transferred to a cropping room at 16 °C, 65–75% RH with 8 h of light daily to induce fructification of the mushroom.

### Substrate evaluation

For each treatment, physicochemical properties and nutrient content of the substrate were analyzed before spawning and after harvesting the mushroom. The substrates were dried at 80 °C to a constant weight in an oven (Contherm Thermotec 2000 oven, Contherm scientific LTD, New Zealand), and subsequently ground into fine powder. Total carbon (C) content was determined according to Walkley and Black^25^. Quantification of total nitrogen (N) content was carried out after hot digestion of 0.1 g substrate powder with conc. sulfuric acid (H_2_SO_4_) following the Kjehldal method^26^. The C:N ratio of each substrate was determined. Organic matter (OM) content was examined according to Allison method^27^. Substrate moisture content was determined according to AOAC^28^, based on the difference between wet and dry weight of the substrate (before and after oven at 70 °C for 72 h). The substrate pH and electrolyte conductivity (EC) were determined according to the methods of Cavins et al.^29^ using a pH and EC meter, respectively. Briefly, sample extraction was performed by mixing 5 g of substrate powder in 50 mL of distilled water. The sample was mixed and saturated by shaking for 10 min and left at room temperature for 1 h. All the extracts were filtered before the measurements. The percent change in value of C, N, C:N ratio, pH and EC was calculated as [(Final Val–e-Initial Value)/Initial Value] × 100, where Final Value is the value after harvesting mushroom, Initial Value is the value before spawning.

### Evaluation of production parameters

The Yamabushitake mushroom was grown and harvested at 47 days after spawning. The number and growth parameters of the fruiting body, including fresh weight (FW) and dry weight (DW), were measured using a digital scale. DW was obtained after drying the fruiting body in an oven at 70 °C for 72 h^30^. Biological efficiency (BE), an estimation of the mushroom ability to convert substrate into fruiting bodies, was calculated by dividing the total fruiting body FW harvested from the whole crop by the total substrate DW. The data was presented as percent BE^31^.

For each substrate treatment, the quality of Yamabushitake mushroom was evaluated after harvesting. The moisture content was calculated as percentage, as previously described by AOAC^28^: FW of sample/DW of sample) × 100. The fruiting body cap diameter was measured and expressed as unit in cm. The number of fruiting body was determined based on the cracks formed on fruiting body at the mature stage^30^.

### Statistical and data analysis

The study was performed in a completely randomized design (CRD), with four biological replicates per treatment. Each biological replicate contains 50 mycelium bottles. All parameters were subjected to analysis of variance (ANOVA). The difference between the means of individual treatments was assessed using Duncan’s multiple range tests (DMRT)^32^ at the significance level of 95%. The results are shown as mean ± standard error (SE). All statistical analyses were performed using the software of Statistical Product and Service Solutions (SPSS, IBM Corporation; Armonk, NY, USA). Determination of the Pearson correlation coefficient between measured variables was performed in R (version 4.1.0), using the corrplot package^33^ for visualization of the correlation matrix. Principal component analysis (PCA) of the parameters was conducted on scaled data and visualized using the ggfortify package^34^.

### Ethical approval

The present study complied with the international and national guidelines for the use of experimental plant material.

## Results

### Rubber and bamboo sawdust constitute mushroom substrates with distinct physicochemical properties

**Figure 1:**
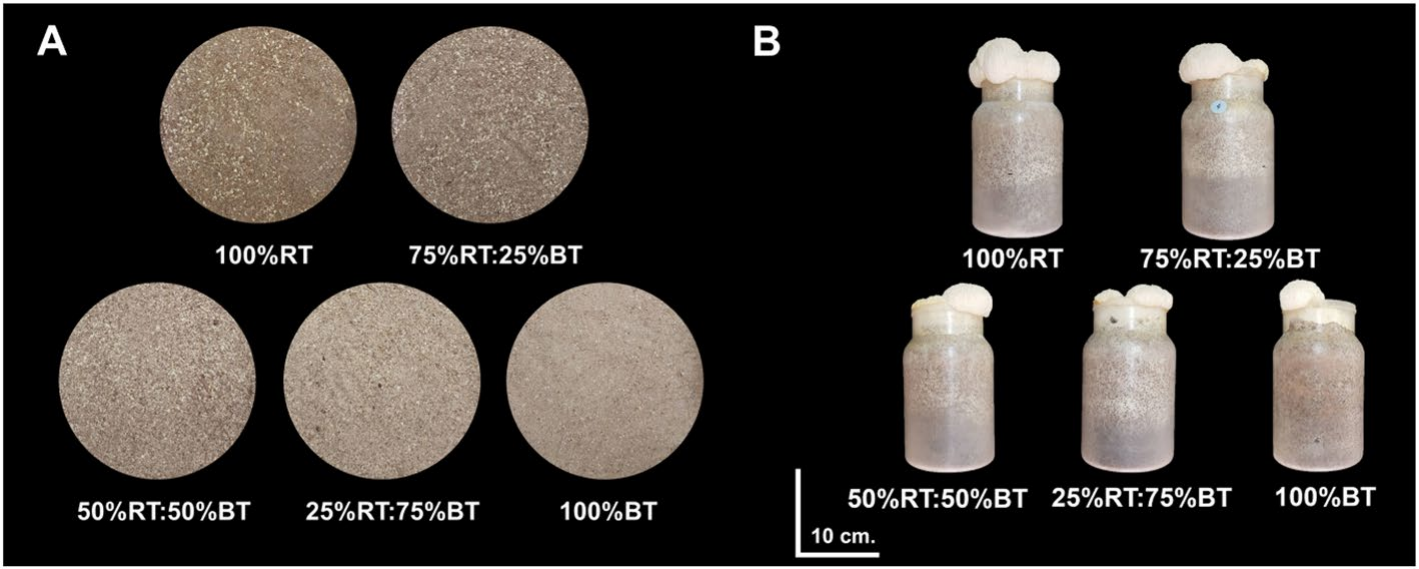
Comparison of different substrate types/ratios (**A**) and growth characteristics of Yamabushitake mushroom grown on each substrate at 40 days after cultivation (**B**). 100%RT, whole rubber sawdust; 75%RT:25%BT, rubber sawdust and bamboo sawdust at the ratio 3:1; 50%RT:50%BT, rubber sawdust and bamboo sawdust at the ratio 1:1; 25%RT:75%BT, rubber sawdust and bamboo sawdust at the ratio 1:3; 100BT%, whole bamboo sawdust.

Physicochemical properties of cultivation substrates known influence mushroom growth, including moisture content, pH and EC, as well as the content of key nutrients were analyzed before spawning (non-inoculated substrates) and/or after harvesting the mushroom (Table 1 and Fig. 1). These were compared between the substrates with different sawdust ratios, including 100% rubber or bamboo sawdust (100%RT or 100%BT, respectively) and those containing rubber and bamboo sawdust mixture at the ratios 3:1 (75%RT:25%BT), 1:1 (50%RT:50%BT), and 1:3 (25%RT:75%BT).

**Table 1:** Chemical composition of the growing substrates before spawning and after harvesting the mushroom as affected by different substrate types/ratios under commercial cultivation.

Treatment	Moisture (%)	pH			Electrical conductivity (dS/m)		
		Before	After	Difference (%)	Before	After	Difference (%)
100%RT	66.81 ± 0.50	6.70 ± 0.03b	4.79 ± 0.03b	− 20.49 ± 0.74b	1.28 ± 0.07b	1.02 ± 0.03b	19.45 ± 3.23b
75%RT:25%BT	67.15 ± 0.17	6.74 ± 0.04b	4.85 ± 0.10b	− 28.02 ± 1.84c	1.14 ± 0.03b	1.23 ± 0.01a	8.68 ± 4.28a
50%RT:50%BT	67.09 ± 0.65	6.89 ± 0.03a	4.87 ± 0.01b	− 29.27 ± 0.41c	1.19 ± 0.07b	1.06 ± 0.03b	− 9.38 ± 7.05b
25%RT:75%BT	66.88 ± 0.62	6.70 ± 0.03b	4.86 ± 0.07b	− 27.47 ± 1.40c	1.31 ± 0.08b	1.23 ± 0.02a	− 5.42 ± 5.43ab
100%BT	66.86 ± 0.68	6.76 ± 0.04b	6.69 ± 0.25a	− 0.89 ± 3.90a	1.96 ± 0.02a	1.00 ± 0.04b	− 48.75 ± 1.99c
F-test	ns	*	*	*	*	*	*

Data are represented as mean ± SE (*n* = 4) with 50 mycelium bottles in a replication. Different letters in the same column indicate significant difference between the treatments according to DMRT at *P* < 0.05. *Indicates significant difference based on F-test. “ns” indicates no significant difference.

The substrate moisture content was measured before spawning. Different sawdust types/ratios did not significantly affect moisture content in the mycelium bottle, with the values grouping around 66.81 and 67.15% (Table 1). On the contrary, the sawdust types and ratios seemed to influence pH and EC of the substate before spawning, as there was a significant difference for these parameters between some of the treatments. Before cultivation, most of the treatments exhibited similar substrate pH (6.0–6.89), except for the 50%RT:50%BT treatment that had a significantly higher pH of 6.89 (Table 1). Mushroom cultivation led to a reduction in the substrate pH in all treatments, but this was less pronounced in the 100% BT treatment as the pH after harvest was approximately 6.69, while the other treatments showed a pH lower than 5. Across the five treatments, the substrate EC before spawning ranged from 1.14 to 1.96 dS/m (Table 1). The highest EC was detected in the 100%BT substrate (1.96 dS/m). After mushroom cultivation, the maximum EC of 1.23 dS/m was achieved for the 75%RT:25%BT and 25%RT:75%BT treatments, while the remainders had EC of about 1.02–1.06 dS/m. Comparison of the substrate EC after harvest with that before cultivation indicated that only the 75%RT:25%BT treatment exhibits an increment of EC over the course of mushroom cultivation. The other treatments resulted in a decrease in substrate EC (Table 1). Overall, these data indicate that sawdust types affect physical properties of the cultivation substrate, which may be relevant to Yamabushitake mushroom production.

**Figure 2:**
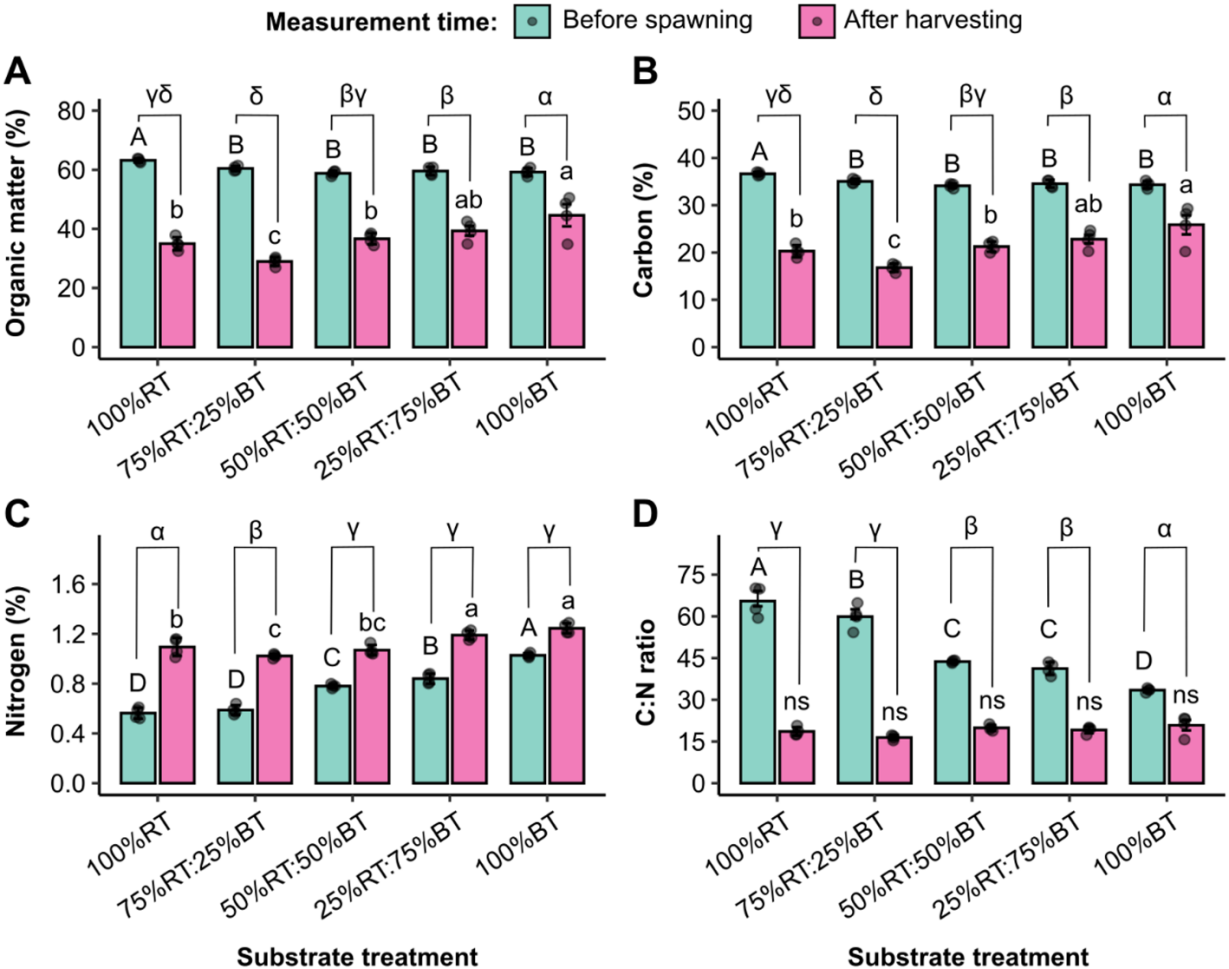
Comparison of nutrient content between substrate types/ratios. The content of organic matter (**A**), carbon (**B**), nitrogen (**C**), and C:N ratio (**D**) are compared between substrate treatments before spawning and after harvesting. Values are represented as mean ± SE (*n* = 4). Different upper-case and lower-case letters above the bars indicate significant differences at *P* < 0.05 across all the treatments before spawning and after harvesting, respectively. Different Greek alphabets show a significant difference in percent change of substrate components at *P* < 0.05. ANOVA was performed, followed by mean comparison with DMRT. “ns” indicates no significant difference. We have presented every single data point in our plots.

For nutrient availability, the OM, C and N content and C:N ratio also varied by the sawdust types/ratios. Before spawning, the OM and C content in the 100%RT substrate was highest at 63.2% and 36.7%, respectively, while these were not significantly different between the other four sawdust types/ratios (Fig. 2A, B). After harvesting, the highest levels of OM and C were retained in the 100%BT and 25%RT:75%BT substrates, whereas the 75%RT:25%BT substrate contained the least amount of OM and C. As a result, a significant degree of the reduction in OM and C content after harvesting relative to the content before spawning was observed in the 100%RT and 75%RT:25%BT treatments (Fig. 2A, B), indicating a better utilization of OM and C by the fungus from these substrates.

Interestingly, unlike OM and C that were most highly enriched, the 100%RT substrate, along with 75%RT:25%BT, showed a significantly lower content of N (at 0.56% and 0.59%, respectively) before spawning, as compared to the other substrates. The N content was highest in the 100%BT substrate (1.03%), with the amount of N generally increasing proportional to the ratio of bamboo sawdust (Fig. 2C). All the treatments showed an increase in N content after harvesting compared to the amount of N before spawning. The greatest increment of N was observed in the 100%RT substrate followed by that of the 75%RT:25%BT treatment, while the degrees of N content augmentation in the other three substrates were not significantly different from one another (Fig. 2C). Notably, of all the five substrate treatments, the 75%RT:25%BT substrate displayed the lowest content of OM, C and N after harvest (Fig. 2A–C). By contrast, the higher ratio of bamboo sawdust was associated with an increased retention/accumulation of OM, C and N in the substrate after harvest. Altogether, these data indicate that rubber sawdust imparts higher content of OM and C while bamboo sawdust increases the content of N to the substrate, and that the utilization of OM and C was most evident in the 100%RT and 75%RT:25%BT substrates.

Next, the C:N ratio of each substrate was evaluated, and it was found that the 100%RT treatment displayed the highest C:N ratio (65.48 ± 2.68) before spawning (Fig. 2D). Like what observed with OM and C content, the 100%BT treatment showed the lowest level of C:N ratio, which was about 33.44 ± 0.37. The change in C:N ratio in the three BT-RT mixture treatments was negatively associated with the percentage of BT, ranging from 59.90 ± 2.18 in the 75%RT:25%BT treatment to 41.21 ± 1.15 in the 25%RT:75%BT treatment. After harvest, all the five treatments showed a similar C:N ratio between 16.43 ± 0.4 and 20.84 ± 1.78, which were all significantly lower than the values before spawning. However, the greatest decrease in C:N ratio was detected in the 100%RT and 75%RT:25%BT treatments (Fig. 2D), suggesting that Yamabushitake mushroom can better utilize the nutrients from these substrates.

### The highest mushroom yield is obtained from the 100%RT and 75%RT:25%BT substrates

**Figure 3:**
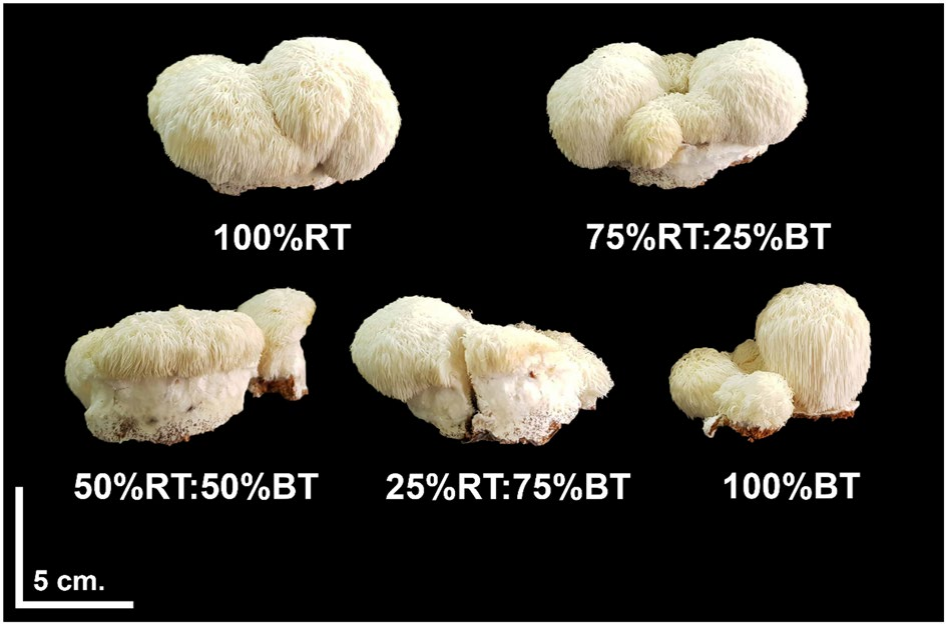
The fruiting bodies of Yamabushitake mushroom grown on different substrate types/ratios harvested at 47 days after spawning.

**Figure 4:**
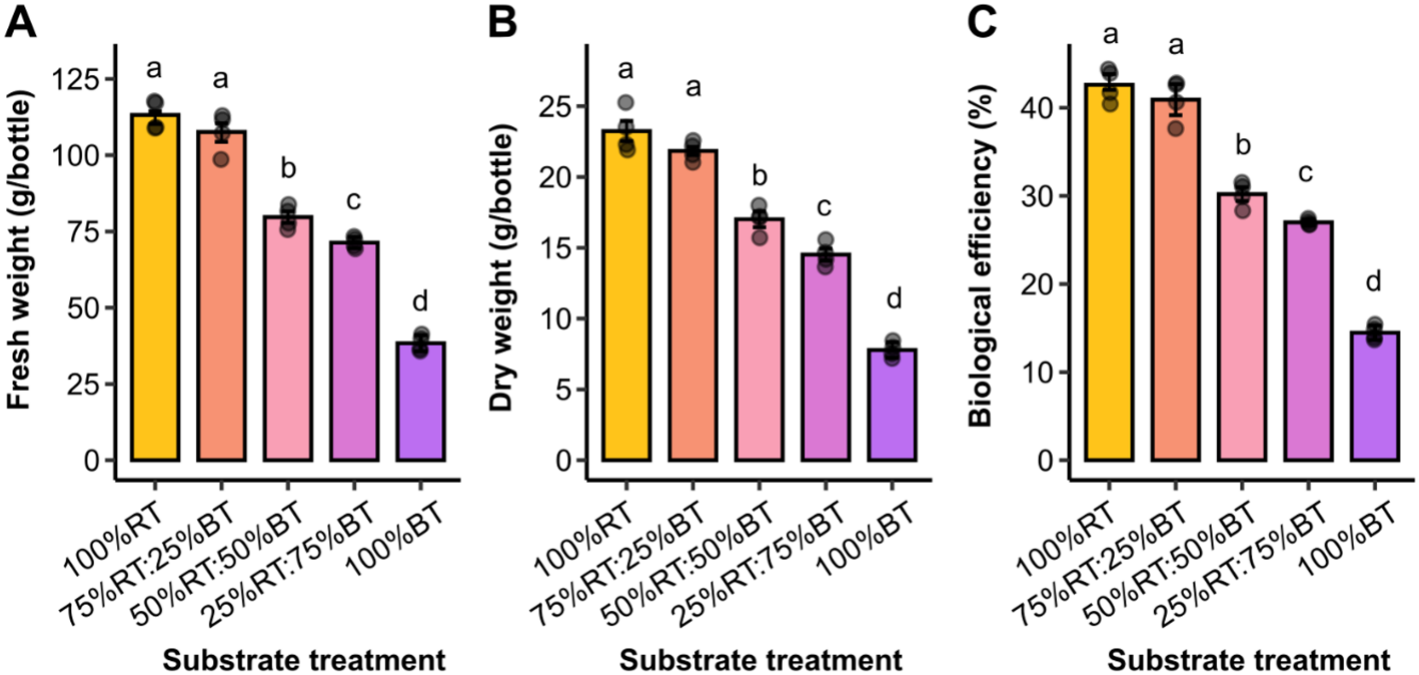
The quantity of Yamabushitake mushroom harvested at 47 days after spawning. Parameters related to mushroom productivity including fresh weight (**A**), dry weight (**B**) and biological efficiency (BE) (**C**) of Yamabushitake mushroom are compared between the five treatments. Bars are represented as mean ± SE (*n* = 4). Different letters above the bars indicate significant difference (*P* < 0.05). ANOVA was performed, followed by mean comparison with DMRT. We have presented every single data point in our plots.

The results of substrate types/ratios on Yamabushitake mushroom production were evaluated at 47 days after spawning (Fig. 3). Parameters related to the quantity and quality of the mushroom fruiting bodies obtained from different treatments were compared. For mushroom quantity, FW, DW and BE were considered. Total fresh mushroom production varied significantly between treatments, from 38.6 to 113.22 g per mycelium bottle. The highest mushroom yield was obtained from the 100%RT substrate treatment, which yielded approximately 113.22 ± 2.43 g FW and 23.25 ± 0.75 g DW of the fruiting body per bottle (Fig. 4A, B). Nevertheless, this was not significantly different from that of the 75%:25%BT treatment, where the average fruiting body FW and DW weight was about 107.62 ± 3.24 and 21.84 ± 0.34 g per bottle, respectively. The other three treatments (50%RT:50%BT, 25%RT:75%BT and 100%BT) resulted in a significantly lower fresh and dry mushroom yield, compared to the 100%RT and 75%:25%BT treatments, with the 100% BT treatment having the least fresh and dry fruiting bodies of 38.36 ± 1.32 and 7.794 ± 0.27 g per bottle, respectively (Fig. 4A, B).

Considering the efficiency of mushroom cultivation by each treatment using BE, a measurement for the conversion of substrate dry weight into mushroom fruiting bodies, it was found that the 100%RT and 75%:25%BT treatments similarly provided the highest BE of 42.61 ± 0.93 and 40.92 ± 1.20%, respectively (Fig. 4C). A trend similar to FW and DW was observed for the other treatments, where BE was reduced in proportion to the increase in bamboo sawdust ratio, with the 100% BT treatment showing the lowest BE of 14.47 ± 0.40% (Fig. 4C). Collectively, it can be concluded that the ability of Yamabushitake mushroom to increase fruiting body production depends on the proportion of rubber sawdust in the substrate.

**Figure 5:**
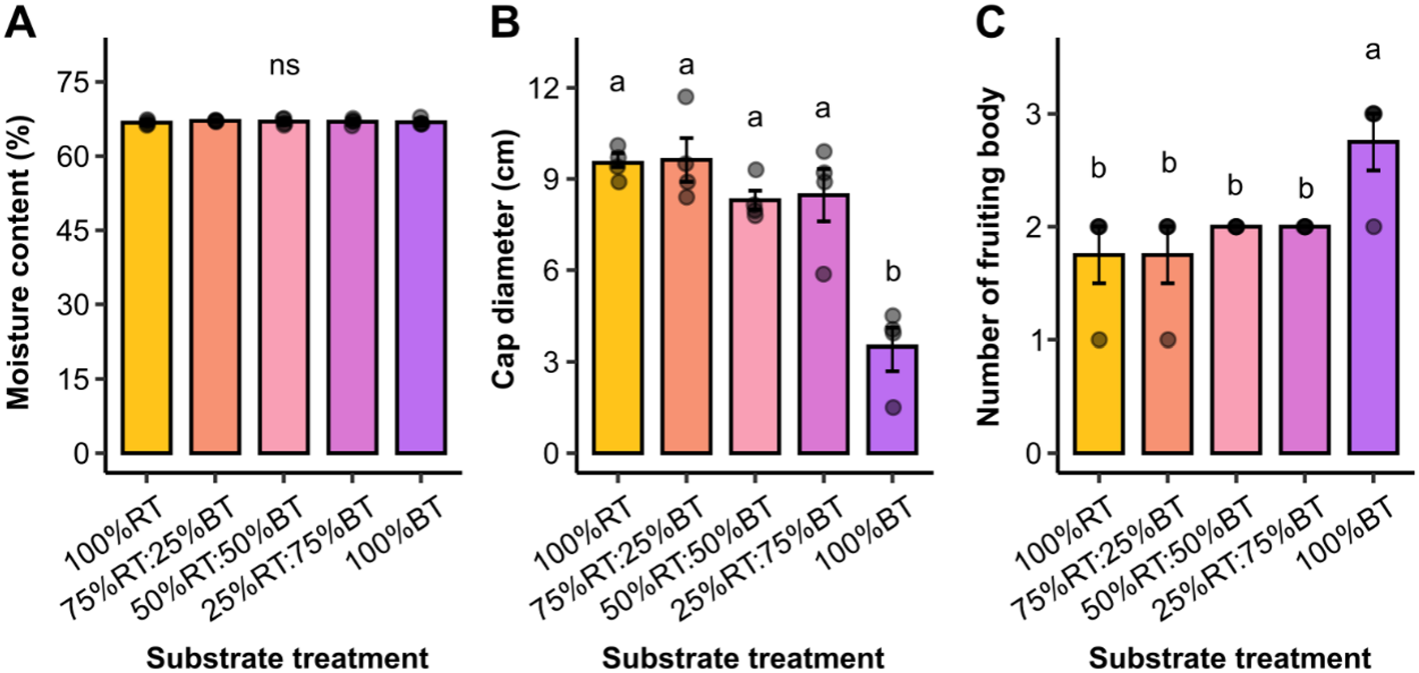
The quality of Yamabushitake mushroom harvested at 47 days after spawning. Mushroom quality attributes including moisture content (**A**), cap diameter (**B**), and number of fruiting bodies (**C**) are compared between the five substrate treatments. Bars are represented as mean ± SE (*n* = 4). Different letters above the bars indicate significant difference (*P* < 0.05). ANOVA was performed, followed by mean comparison with DMRT. “ns” indicates no significant difference. We have presented every single data point in our plots.

For mushroom quality, the moisture content, cap diameter, and the number of fruiting bodies were evaluated. While there was no significant difference in the moisture content of mushroom products (Fig. 5A), cap diameter and the number of fruiting bodies were markedly different in one treatment. Mushroom cap diameter was found highly similar for the treatments 100%RT, 75%RT:25%BT, 50%RT:50%BT and 25%RT:75%RT (8.47–9.63 cm), but it was significantly reduced to about 3.50 ± 0.68 cm in the 100%BT treatment (Fig. 5B). This was concordant with the quantitative parameters, total biomass and BE. On the other hand, the 100%BT treatment exhibited a significantly greater number of fruiting bodies than other treatments, at 2.75 ± 0.25 per mycelium bottle (Fig. 5C). These data indicate a trade-off between the size and number of Yamabushitake mushroom fruiting bodies for the 100%BT treatment, where 100% bamboo sawdust substrate results in the mushroom of a smaller size but with higher number of the fruiting body.

### Principal component analysis reveals key factors associated with mushroom yield and quality

**Figure 6:**
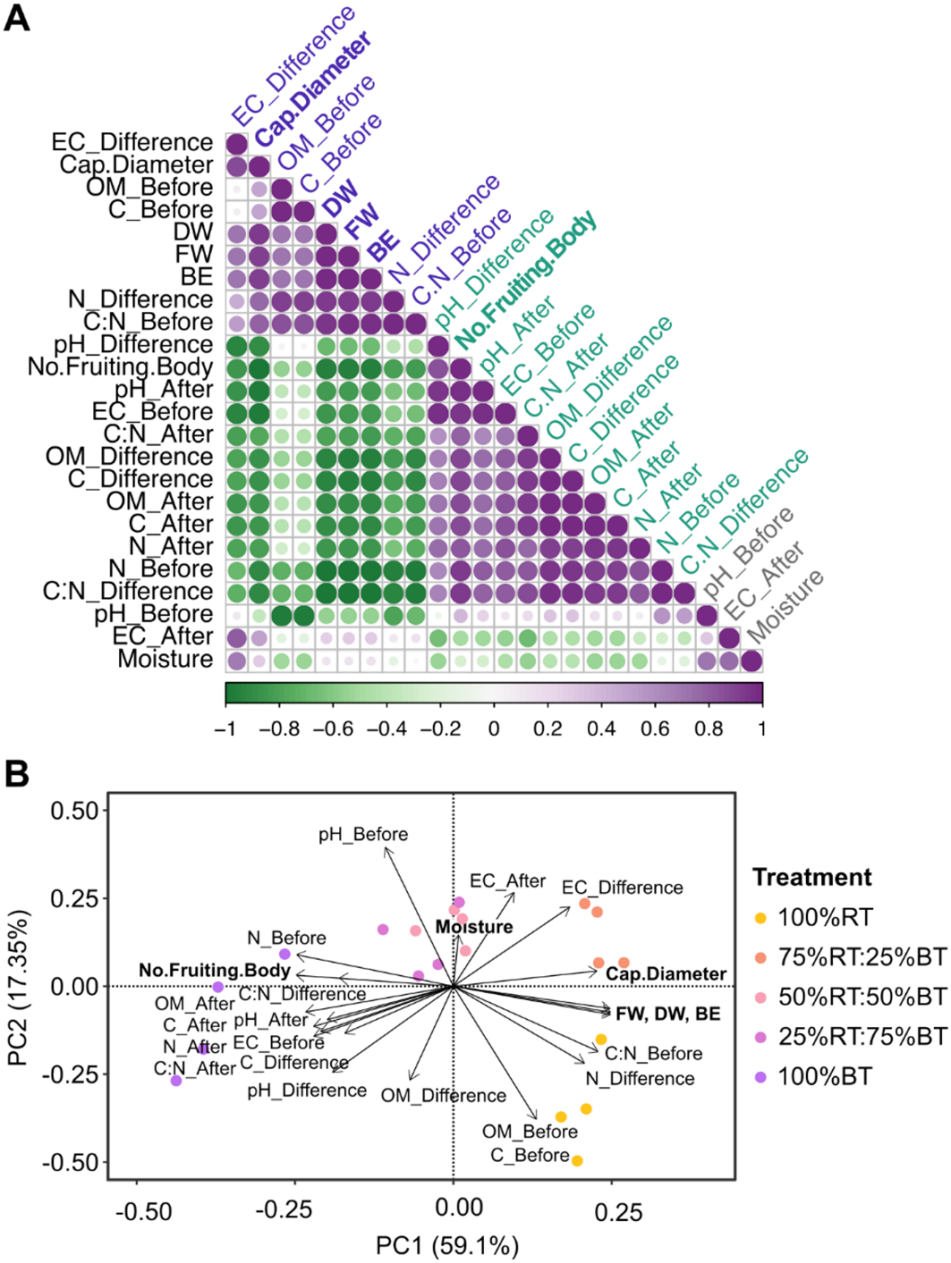
Correlation plot (**A**) and PCA biplot (**B**) of parameters related to yield and quality of Yamabushitake mushroom and physicochemical properties and nutrient content of the substrate. The analysis includes six variables that represent mushroom growth and quality, including fresh weight (FW), dry weight (DW), biological efficiency (BE), cap diameter (Cap.Diameter), moisture content (Moisture), and number of the fruiting body (No.Fruting.Body). 18 variables represent the physicochemical properties of the substrates, including organic matter content (OM), carbon content (C), nitrogen (N), C:N ratio (C:N), pH (pH) and electrical conductivity (EC), which were measured before spawning (Before) and after harvesting (After), and the change of each variable between the two time points (Difference or Diff.). Color scale in (**A**) indicates the Pearson correlation coefficient, with the variables ordered based on hierarchical clustering. We have presented every single data point in our plots.

To understand the overall trend whether and how the yield and quality of Yamabushitake mushroom correlates with substrate physicochemical properties and nutrient contents, Pearson correlation analysis was conducted on all measured variables for the five substrate types/ratios. A strong and significant positive correlation was detected for the yield parameters including FW, DW and BE with the C:N ratio before spawning (*r* = 0.96–0.97, *P* < 0.01) and the change in N content after cultivation (*r* = 0.92–0.94, *P* < 0.05), while these were negatively correlated with N content before spawning (*r* = − 1, *P* < 0.001), the change in OM and C content and C:N ratio (*r* < − 0.94, *P* < 0.01) (Fig. 6A). For mushroom quality, a correlation between cap diameter and nutrient content of the substrate was consistent with those of yield parameters, albeit to a lesser degree and levels of significance. However, the number of fruiting bodies was negatively correlated FW, DW, BE and mushroom cap diameter (*r* < -0.95, *P* < 0.01), but showed a positive correlation with substrate EC and N content before spawning (*r* = 0.93–0.95, *P* < 0.05), pH after harvesting (*r* = 0.96, *P* < 0.01), and the change in OM and C content and C:N ratio after cultivation (*r* < 0.89–0.93, *P* < 0.05) (Fig. 6A).

To investigate a clustering pattern among the five substrate treatments in Yamabushitake mushroom cultivation and identify key features contributing to the variation between the treatments, principal component analysis (PCA) was performed on all replicate parameters that represent both physicochemical properties and nutrient content of the substrates and mushroom growth and quality. As presented in Fig. 6B, the PCA biplot indicates that the two most important principal components (PC) accounted for 76.45% of the variance. The first PC described 59.1% of the variation, and this clustered the substrate treatments into three groups. The first group included the 100%RT and 75%RT:25%BT treatments, while the 50%RT:50%BT and 25%RT:75%BT substrates were clustered together as group 2, which was separated from the 100%BT treatment in group 3 (Fig. 6B). PC1 was positively contributed by the mushroom quantity (FW, DW and BE) and cap diameter, together with C:N ratio before spawning, the change in EC and N content after cultivation, and to a lesser extent with the OM and C content before spawning. This was corroborated by the observation that the increase in these parameters was a hallmark of the 100%RT and 75%RT:25%BT treatments. On the other hand, a strong negative correlation on PC1 was observed for the number of mushroom fruiting bodies, together with N content and EC before spawning, the content of OM, C, N, C:N ratio and substrate pH after harvesting, and the change in pH, C content and C:N ratio after cultivation (Fig. 6A), in line with their reduction in the 75%RT:25%BT and 100%RT treatments but pronounced increase in the 100%BT treatment. The second PC covered 17.35% of the variation and separated most of the 100%RT replicates from the rest of substrate types/ratios (Fig. 6A). PC2 was negatively driven by the content of OM and C before spawning, which was associated the 100%RT substrates having higher OM and C than the other treatments.

## Discussion

In this study, different ratios of rubber and bamboo sawdust were tested for Yamabushitake mushroom production, and the results revealed that the maximum mushroom yield is obtained from the substrates containing 100% rubber sawdust (100%RT) and 3:1 rubber and bamboo mixture (75%RT:25%BT), whereas 100% bamboo sawdust (100%BT) produces the lowest yield. Mushroom generally obtains nutrients from the substrate at a specific range of pH and EC^35,36^. While mushrooms can grow at a broad pH range from 4.3 to 8.5^37,38^, the optimal pH for mushroom culture appears to be neutral^35^. Our measurement of substrate pH before spawning indicated that rubber and bamboo substrates, either by using alone or as a mixture, provide substrates with the pH of 6.70–6.89, which is considered optimal (Table 1). Of note, while the pH of other substrate treatments decreased after cultivation, that of the 100%BT substrate remained at the similar level. This might be relevant to low mushroom growth in this treatment.

For typical mushroom, optimal growth and development of the mycelium can be achieved at an EC around 0.87–1.98 dS/m^39,40^. In this study, the 100%RT and all the combined RT-BT substrate treatments showed EC levels between 1.02 and 1.31 dS/m, which is deemed optimal (Table 1). These data suggest that rubber sawdust, alone or in combination with bamboo sawdust, has EC suitable for Yamabushitake mushroom production. Nevertheless, this might not be the case for bamboo sawdust at 100% because it constitutes the substrate with a much higher EC before spawning (Table 1). After cultivation, the EC of most substrate treatments significantly increased compared to the value before spawning, which is in accordance with previous reports by Philippoussis et al.^41^ and Öztürk and Atila^42^.

The content of OM and C and the C:N ratio before spawning were greater in the 100%RT treatment, while its N content was the lowest (Fig. 2). The 100%RT treatment and 75%RT:25%BT treatments had the greatest reduction in OM and C content and the C:N ratio during cultivation, when compared with other treatments. This suggests that the nutrients are better utilized from these substrates to support growth of the fruiting bodies, which is corroborated by the higher mushroom yield. Additionally, N accumulation in the substrate after cultivation was associated with a decrease in the ratio of rubber sawdust. This was consistent with the studies by Philippoussis et al.^41^, Gaitan-Hernandez et al.^43^ and Hoa et al.^44^. An overaccumulation of N in the substrate might contribute to the loss of mushroom biomass after solid-state fermentation. Since mushroom requires OM and C over N as the main nutrient source^45,46^, these data revealed 100%RT and 75%RT:25%BT as the most suitable substrate candidates for Yamabushitake mushroom production.

Cultivation of Yamabushitake mushroom on the 100%BT substrate showed a higher number of fruiting bodies, but with smaller cap size and yield (Fig. 5). This could be a result of the sustained alkaline pH of the substrate that might negatively affect mushroom growth and development^47,48^. Indeed, a previous study by Khan et al.^35^ has shown that highly alkaline substrate inhibits mycelial growth by reducing nutrient uptake of Oyster mushroom. Collectively, these results suggest that, in the case of rubber sawdust shortage, the maximum of 25% supplementary bamboo sawdust could be used as an alternative substrate for Yamabushitake mushroom production without a penalty on the mushroom yield and quality.

Mushroom production is a process that involves bioconversion of lignocellulosic materials into nutrients to support mushroom growth^49,50^. A correlation between elemental composition of the substrates and mushroom growth has been demonstrated^51^. This is consistent with our study in Yamabushitake mushroom cultivation, which showed a positive correlation of the parameters reflecting mushroom growth (FW, DW and BE) with the C:N ratio before spawning and the change in N content after cultivation (Fig. 6A). Indeed, PCA supports this notion, with the biplot showing that the 100%RT and 75%RT:25%BT samples were clearly split from the other treatments on PC1, driven by the C:N ratio and OM and C content before spawning and the change in N content and substrate EC after cultivation (Fig. 6B). It remains to be further explored whether other physiocochemical parameters, such as lignin, cellulose, and hemicellulose content, contribute to the higher mushroom growth with rubber sawdust but lower yield with bamboo dust substrates. Based upon the 100%RT or 75%RT:25%BT substrate treatments, future attention will also be paid to improving the cultivation method, such as substrate formulation and containers, to improve yield and production of bioactive compounds from the mushroom.

## Conclusions

This study demonstrates the use of rubber sawdust, an agricultural waste from the rubber industry, for industrial-scale cultivation of Yamabushitake mushroom in a tropical region. The substrates containing 100% rubber sawdust and a mixture of rubber and bamboo sawdust at the ratio 3:1 provide a comparable maximum mushroom yield. Therefore, a substitution of rubber with bamboo sawdust may be viable with regards to the maintenance of mushroom yield and quality, when bamboo sawdust is used at a low ratio, providing that the pH, EC, OM and C content, and C:N ratio of the substate remain in an optimal range. Essentially, this represents an alternative cultivation substrate for Yamabushitake mushroom production when rubber sawdust is in short supply.

## Data Availability

The datasets used and/or analyzed during the current study are available from the corresponding author on reasonable request.
